# The role of CD36 receptor in the phagocytosis of oxidized lipids and AMD

**DOI:** 10.18632/aging.100237

**Published:** 2010-11-28

**Authors:** Yves Courtois

**Affiliations:** FARVO, CRC INSERM 1990, 75016 Paris, France

With the aging of the population in industrialized countries, mechanisms involved in the development of age-related macular degeneration become essential to understand.

AMD is in two forms, a dry or atrophic form and a wet or neovascular form. However, these two forms were characterized in their common early form (also described in aged retina [1]) by the presence of deposits under the retinal pigment epithelium (RPE), in Bruch's membrane (BM; [22]). The origin of drusen is not well established, and several hypothesis are built. Oxidative damage is considered as a major event [3]. Nevertheless, numerous studies using various histological, histochemical and microscopical techniques converge to demonstrate the presence in drusen of esterified and unesterified cholesterol, triglyceride, lipo-proteins [4-7].

In this issue of Aging, Picard *et al.* were interested in the mechanism of drusen formation promoting thickening of BM; resulting in decrease in RPE permeability and therefore a failure to transport nutrients from the RPE to photoreceptors, resulting in theirs deaths [8-9].

Picard et al. confirmed the results already foreshadowed by other teams that the cells of RPE are able to up-take and internalize oxidized lipids (oxLDL) via scavenger receptor CD36 [10]. The same team had previously shown that CD36 deficiency leads to choroidal involution [11]. In this new paper, the authors demonstrated that aged-mice deficient in CD36 have thickening of BM with formation of sub-RPE deposits such as “drusen”. Using a model often used for studies on atherosclerosis, ApoE−/− mice, they confirmed that CD36 deficiency increases BM thickness [12] and correlated this with increased plasma oxLDL level.

So it seems that the CD36 receptor deficiency has a role in the formation of deposits due to lipid excess during aging [13] under RPE, resulting in a breakdown between the bloodstream (choroicapillaries) and RPE-photoreceptors [14,15]. They also demonstrated that this phenomena may be partially restored by stimulating the expression of CD36 with a peptide derived of growth hormone.

To conclude, the study of Picard et al. highlighted the important role of CD36 in maintaining homeostasis of the pigmented epithelium. CD36 deficiency in aging and more severely during the early stage of AMD [16] associated with a cumulative effect of oxidative stress could be one of the primordial players in the development of late stages of the disease.
